# Kinetic Study of Encapsulated β-Carotene Degradation in Dried Systems: A Review

**DOI:** 10.3390/foods11030437

**Published:** 2022-02-02

**Authors:** Vera Lavelli, Jolanta Sereikaitė

**Affiliations:** 1 Department of Food, Environmental and Nutritional Sciences (DeFENS), University of Milan, 20133 Milan, Italy; 2 Department of Chemistry and Bioengineering, Vilnius Gediminas Technical University, 10223 Vilnius, Lithuania; jolanta.sereikaite@vgtu.lt

**Keywords:** β-carotene, encapsulation, water activity, glass transition, kinetics, drying

## Abstract

β-Carotene serves as a precursor of vitamin A and provides relevant health benefits. To overcome the low bioavailability of β-carotene from natural sources, technologies have been designed for its encapsulation in micro- and nano-structures followed by freeze-drying, spray-drying, supercritical fluid-enhanced dispersion and electrospraying. A technological challenge is also to increase β-carotene stability, since due to its multiple conjugated double bonds, it is particularly prone to oxidation. This review analyzes the stability of β-carotene encapsulated in different dried micro- and nano-structures by comparing rate constants and activation energies of degradation. The complex effect of water activity and glass transition temperature on degradation kinetics is also addressed, since the oxidation process is remarkably dependent on the glassy or collapsed state of the matrix. The approaches to improve β-carotene stability, such as the development of inclusion complexes, the improvement of the performance of the interface between air and oil phase in which β-carotene was dissolved by application of biopolymer combinations or functionalization of natural biopolymers, the addition of hydrophilic small molecular weight molecules that reduce air entrapped in the powder and the co-encapsulation of antioxidants of various polarities are discussed and compared, in order to provide a rational basis for further development of the encapsulation technologies.

## 1. Introduction

Vitamin A deficiency is a serious health problem especially in low and middle-income countries and affects approximately 33% of preschool-age children around the world. It is essential for vision, especially dark adaptation, bone growth and reproduction and it maintains integrity of various tissues [1]. Among others, vitamin A plays an essential role in immune system function and is called “the anti-infective vitamin”. Vitamin A deficiency weakens the host response to infection. Those functions of vitamin A are of primary importance in the context of the new infection COVID-19 [2,3].

β-Carotene serves as a precursor of vitamin A. In humans, dietary β-carotene is cleaved to two molecules of all-*trans* retinal by the action of β, β-carotene 15,15′–monooxygenase 1. Subsequently, retinal can be reduced into retinol by a retinal reductase or oxidized into retinoic acid by the action of retinal dehydrogenase [4]. β-Carotene is one of the most potent antioxidants. It not only quenches singlet oxygen but also inhibits lipid peroxidation [5]. β-Carotene reduces the risk of Alzheimer’s disease [6], has a protective effect against cardiovascular diseases [7] and type 2 diabetes [8] and decreases the incidence of blindness [9]. It is also believed to prevent cancer [10]. β-Carotene is found in various fruits and vegetables, while vitamin A is present in food of animal origin [11]. Hence, β-carotene could play a significant role as a source of vitamin A in the perspective of replacing animal-source foods with plant-based foods, in order to address environmental, food security and public health objectives [12]. However, β-carotene is poorly bioavailable from its native vegetable sources [13,14] and it is particularly sensitive to degradation [15,16,17]. In fact, due to its many conjugated double bonds, β-carotene is prone to autooxidation during food processing [15,16,17,18,19,20]. Factors that accelerate β-carotene degradation are temperature, light, oxygen radicals, free radicals formed by lipid peroxidation and transition metal cations [15]. The pathway for β-carotene degradation was proposed to begin with a *cis*–*trans* isomerization that may take place at C9–10 or C13–14 or C15–15′ and is followed by the rupture of the *cis* double bond, with the generation of a diradical molecule with an oxygen bound to just one carbon atom; then, a peroxide intramolecular cycle is formed that triggers the formation of apocarotenoids upon its rupture [17,18]. Some studies demonstrated that β-carotene isomerization is a reversible process and the *cis* isomers can be converted into each other or also return to the all-*trans* form. However, the rate constant of β-carotene degradation into oxidation and cleavage products is an order of magnitude larger than the rate constants of isomerization, indicating that degradation is favored above isomerization [17]. It is worth noticing that the study of the single reaction steps is the only approach to get insight into the actual reaction pathway. On the other hand, the majority of the kinetic models to describe β-carotene degradation in food systems are empirical models of the global process, based on β-carotene decay. Empirical models do not provide insight on the single steps but represent a straightforward and pragmatic approach to study β-carotene decay during food processing [17]. For β-carotene degradation, a first-order kinetics was most often found for the global process and the effect of temperature on the rate constant was found to follow the Arrhenius equation [17].

Given the susceptibility of β-carotene to degradation, technological strategies are searched to improve its stability. A promising approach is extraction of β-carotene from largely accessible sources, mainly carrot and mango peels [21], thus applying the criteria of circular food processing systems [22]. Then, specific micro- or nano-structures are developed to encapsulate β-carotene, in order to improve its bioaccessibility and stability [23]. The stability of encapsulated β-carotene in aqueous environments has been reviewed [24]. Encapsulated β-carotene is also obtained in dried structures since drying is particularly important in view of food distribution within the global food system [25]. The term “dry chain” was introduced to describe food chains where dehydration is applied to food products to prevent microbial growth [25]. Even if drying is a highly energy consuming operation [26], it provides the advantage that no further infrastructures, equipment and energy input are required to maintain product quality during storage and transport.

This review study illustrates the main technologies to produce β-carotene encapsulated in dried matrices. Then, it focuses on the kinetic data of β-carotene degradation (rate constants, activation energy) as affected by the formulation, water activity (aw) and glass transition temperature (Tg) of the matrix. As a comparison, kinetic data on β-carotene degradation in a natural source of β-carotene such as dried carrot is also reported. The aim is to derive knowledge to increase β-carotene stability in the food structures. The choice of selecting a pure carotenoid compound instead of a natural carotenoid-rich extract allowed to understand the behavior of different compounds used for micro- and nano-structure formation.

## 2. Methods

### 2.1. Literature Study

The literature study was performed in the period 2013–2021 using two databases (Scopus and Web of Science). Firstly, the search included the following keywords: “carotene” and “water activity” or “carotene” and “glass transition”. Papers reporting the development of an encapsulation technology for β-carotene and the kinetic study of its stability were selected. Other relevant papers were found among those cited in the papers published from 2013 to 2021. In order to compare the kinetic data obtained by various authors, the stability of the system under dark conditions was only considered since the effect of light exposure was reported only in a few studies, with different lighting conditions applied, thus excluding the opportunity of comparison.

### 2.2. Modelling

In most of the studies the kinetic for β-carotene degradation was fitted to a first-order kinetics as following Equation (1) [17],

Ct/Co = exp(−k × t)(1)

where Ct is β-carotene concentration at time t, Co is the initial concentration of β-carotene, t is the storage time, k is the degradation rate constant at the temperature considered.

A special case of first-order model is the fractional conversion model, which can be applied when a fraction of compound under study remains after the kinetic study and is expressed as following Equation (2) [27]:

(Ct − C_∞_)/(Co − C_∞)_ = exp(−k × t) (2)

where Ct, Co, k and t are the same as in Equation (1), C_∞_ is the asymptotic value to which the retention approaches at a very long time; if C_∞_ ⟼ 0 the equation tends to a first-order kinetic model.

Alternatively, the Weibull model was applied as following Equation (3) [27]:

Ct/Co = exp[−(k × t)^n^] (3)

where Ct, Co, k and t are the same as in Equation (1) and n is a shape factor. The Weibull model is flexible owing to the inclusion of the shape constant in addition to the rate constant. If the degradation rate increases over time then the shape factor is greater than 1, if the degradation rate decreases over treatment time then the shape factor is less than 1 and if degradation rate is constant over time the shape factor is 1 (i.e., first-order kinetics) [27].

If not indicated by the original paper, the half-life of β-carotene was interpolated by the graphs in the study reporting the time course of degradation during storage. Alternatively, the half-life (t_1/2_) was calculated as the following Equation (4):

t_1/2_ = (0.693)^^(1/n)^/k (4)

where t_1/2_ is the half-life; k is the same as in Equations (1)–(3) and n is the shape factor as indicated in Equation (3) or n = 1 in Equation (1).

To calculate the activation energy, if not reported in the original paper, the Arrhenius equation was used, as following Equation (5):

ln(k_1_/k_2_) = (1/T_2_ − 1/T_1_) × Ea/R (5)

where k_1_ and k_2_ are the rate constants at the temperatures T_1_ and T_2_ (Kelvin), respectively, Ea is the activation energy (J/mol), R is the universal gas constant (8.314 J × K^−1^ × mol^−1^) [17].

## 3. Results

### 3.1. Technologies for β-Carotene Encapsulation in Dried Systems

The preliminary step for β-carotene encapsulation is the extraction from natural sources. Due to its high hydrophobicity, β-carotene is extracted using organic solvents such as hexane, acetone, methanol and ethanol, as well as various solvent combinations [11]. Efficient and sustainable approaches have also been developed for β-carotene extraction, based on the application of pulsed electric fields [28], microwaves [29,30], ultrasounds [31], supercritical fluids [32,33] and pressurized liquids [34,35]. β-carotene was also extracted from carrot peel waste by a green procedure without any solvent, based on water-induced hydrocolloidal complexation with pectin [36]. Then, various of micro- and nano-structures designed for β-carotene encapsulation, such as inclusion complexes, single-layer and multilayer oil-in-water (O/W) emulsions, as well as double water-in-oil-in-water (W_1_/O/W_2_) emulsions, are produced in liquid phase and dried [37,38].

Freeze-drying is often applied to dehydrate pre-formed micro- and nano-structures that encapsulate β-carotene. Freeze-drying is characterized by three steps: (a) freezing (b) primary drying that occurs below the triple point (at 0.01°C and 0.612 kPa) when ice is sublimated; and (c) secondary drying, when the remaining unfrozen/bound water is desorbed from the drier food matrix [39]. Parameters which describe the freeze-drying process are freezing temperature and rate, working pressure, shelf temperature (30–50 °C) and processing time [40]. In most studies these parameters are not specified. Freezing temperature and rate are important for the formation and size of ice crystals. Indeed, fast freezing rate produces small ice crystals. The ice crystal size affects heat and mass transfer properties during dehydration as well as porosity and rehydration of the dried product [28]. The sublimation process is strongly affected by pressure, which determines the ice sublimation temperature. To avoid the loss of product structure, during the primary drying the temperature should be below the collapse temperature, which is generally 2 to 20 °C higher than Tg. The shelf temperature is also important since in the secondary drying the product temperature increases to approach that of the shelves. Whatever the processing conditions, freeze-drying is considered to cause little damage to bioactive compounds and to the product structure [39]. On the other hand, the porous structure of the obtained powders, which is desirable for rehydration, could affect the stability of encapsulated bioactive compounds during storage since the presence of micropores increases the contact area that allows the diffusion of oxygen through the polymer matrix and thus decreases the oxidative stability of freeze-dried products [41]. Moreover, freeze-drying has the drawback to be the most expensive process for manufacturing a dehydrated product, having 4 to 10 times more energy requirements than hot air drying [39].

Spray-drying has also been used as an alternative to freeze-drying to obtain encapsulated β-carotene in dried matrices. As for other lipophilic bioactive compounds, in a first step micro- or nano-encapsulation of β-carotene is obtained in the aqueous phase, then spray-drying is applied [42]. Spray drying consists of three steps: atomization of feed sample, drying of liquid droplets and powder recovery [43]. During atomization, liquid feed is passed through an atomizer to the drying chamber and distributed into tiny liquid particles. In fact, the atomization maximizes the surface volume area of liquid feed for efficient drying. The properties of the final product depend on the formulation and combination of different processing conditions, which include feed concentration, inlet temperature (110–220 °C), feed flow rate (2–600 mL/h), drying air flow rate, type of atomizer and atomizer speed [43]. These conditions affect retention of bioactive compounds and also the size and shape of the powder. In general, small particles are desirable because larger particles might be hollow inside and have a less compact structure, which leads to relatively low bulk density compared to fine particle. Spray drying generally produces powders with higher bulk density than freeze-drying, i.e., with little micropores, which is positive because it reduces packaging and shipping costs and increases flowability [44]. Spray-drying is more economic than freeze-drying but it can cause loss of β-carotene during processing due to the high temperatures applied and large contact surface with air. Despite the fact that spray-drying has been widely used for carotenoid encapsulation, different results have been described and optimal processing conditions are still to be identified.

Supercritical fluid technology has also been employed to encapsulate β-carotene-rich oils through solution-enhanced dispersion (SEDS). In general, the components to be encapsulated by SEDS are initially dissolved in a suitable organic solvent by vigorous mixing, and then they are rapidly sprayed along with the supercritical fluid through a coaxial nozzle into a high-pressure vessel, which generates small-sized droplets. Then, the mass transfer of the solvent to the supercritical fluid at a high rate results in the formation of small-sized particles. Factors that affect processing efficiency are temperature, pressure, supercritical fluid used as well as organic solvents (if used) and their flow rates, as well as the component nature and concentrations [45]. Among all the supercritical fluids available, supercritical CO_2_ (SC-CO_2_) has attracted enormous interest due to its wide adaptability, safety, cost-effectiveness, and requiring mild conditions for operation (temperature 31.1 °C and pressure 73.8 bar) [46]. The challenge in this technique is to replace the use of organic solvent with water. In fact, water has a low solubility in SC-CO_2_ [33] in comparison with organic solvents commonly used for SEDS. Hence, the use of O/W emulsions in the place of solvent requires a process setup in order to improve water solubility in SC-CO_2_. For instance, the water removal efficiency can be raised by increasing the pressure and temperature [47].

Electrospraying has also been proposed for β-carotene encapsulation. Electrospraying is an electrohydrodynamic processes, where a polymer solution is sprayed by the application of high potential electric field to obtain particles [48]. The typical setup for electrospraying consists of four main components: (i) a high voltage source (1–30 kV); (ii) a blunt ended stainless steel needle or capillary; (iii) a syringe pump; and (iv) a grounded collector either flat plate or rotating drum. The process is run at ambient temperature [36]. The first step of lipophilic compounds encapsulation consists in the preparation of a O/W emulsion. The emulsion is atomized by electrical forces into highly charged droplets that are self-dispersing in space, thereby preventing droplet agglomeration and coagulation. Further, evaporation of the solvent leads to contraction and solidification of droplets resulting in solid polymeric particles deposited on the grounded collector. Parameters that affect the process include electrical potential applied, flow rate of the solution and distance between the tip of the needle and the collector [49].

Overall, drying processes could affect the stability of β-carotene. In fact, exposure to air and high temperatures could promote chemical degradation, while heating, freezing and shear-stress during processing could cause the rupture of the pre-formed micro- and nano-structures, releasing β-carotene, which becomes exposed to degradation. Moreover, the drying process affects the number and size of the pores of the powder, which entrap oxygen. Hence, encapsulation efficiency and bulk density are relevant parameters for powder stability and they will be considered in the following analysis of β-carotene degradation rate, when available.

### 3.2. Water Activity and Glass Transition Temperature Effect on Dried Product Stability

Drying of β-carotene encapsulated into micro- and nanostructures should reach a final aw level at which matrix stability is maximum. In fact, aw is a thermodynamic parameter that affects the rate of microbiological, chemical and physical phenomena [50]. There is a threshold water activity below which no microorganism can grow, which varies slightly depending on the composition of food matrix. Pathogenic bacteria cannot grow below aw of 0.85, whereas yeasts and molds are more tolerant to reduced aw, but usually no growth occurs below aw of about 0.6 [51]. Conversely, both chemical and physical modifications can occur in dried foods over a wide aw range with different rates, affecting the stability of bioactive compounds. β-carotene degradation follows an oxidative pathway that is triggered by lipid oxidation and transition metal cations. Lipid oxidation in dried foods generally displays a U-shaped curve with respect to aw level. In fact, minimum oxidation rate for lipids occurs when sample moisture is near the Guggenheim–Anderson–de Boer (GAB) monolayer value, which typically is when aw is between 0.2 and 0.4; above and below this range lipid oxidation rate increases remarkably [52]. The decrease of oxidation rate that occurs with change from the anhydrous state to intermediate moisture values (0.2–0.4) has been attributed to multiple factors: the hydration of transition metal cations, rendering them less active promoters of autoxidation; the movement of lipid hydroperoxides to oil–water boundaries and their exclusion from the propagation reactions due to hydrogen bonding with water; the free radical termination reactions due to the movement of polar radicals at the oil-water interfaces. With increase of aw from intermediate values to above 0.7, lipid oxidation rate increases again owing to the increased mobility of transition metal cations and the decreased viscosity. Further increase in aw decreases unsaturated lipid degradation because of the dilution of reacting species. It is worth noting that the specific aw range at which oxidation occurs at the lowest rate is difficult to predict as it depends on the matrix composition [52].

The Tg is another thermodynamic parameter that affects food stability. Tg is the temperature above which a hard amorphous solid will transform into a soft, rubbery material. The Tg depends on matrix composition as well as on the moisture content. In fact, as water content increases, Tg decreases; thus, Tg also decreases with increasing aw [52,53,54]. Materials in the glassy state have very high viscosity and heating to T > Tg results in a marked reduction of viscosity and increase in molecular mobility. In general, in the glassy state various chemical reactions may become diffusion limited and foods are stable as compounds involved in deteriorative reactions take many months or even years to diffuse over molecular distances and approach close enough to each other to react [52,53,54]. In fact, the rate of some reactions increases as the difference between the storage temperature (T) and Tg increases [52,53,54]. However, β-carotene degradation occurs via an oxidative pathway and oxygen, being a small molecule, can diffuse within glassy solids [53]. Accordingly, oxidative processes have been observed to occur at sub-Tg temperatures [54,55]. Oxygen was found to permeate from atmospheric air into oil encapsulated in a glassy matrix (a food model made from sucrose, maltodextrin and gelatin) [56]. Interestingly, in this latter system, a fraction of oil was found to oxidize rapidly and another fraction of oil was found to oxidize more slowly, due to inhomogeneity in the degree of encapsulation. Hence, the efficiency of encapsulation and the oxygen barrier properties of the encapsulation system are fundamental to prevent β-carotene oxidation [56]. Moreover, the rate of oxidation is related to the collapse phenomenon [52,57]. In fact, at T > Tg, the material undergoes structural collapse, which is defined as the readily visible deformation (or shrinkage) of the sample under the force of gravity during storage of a dried amorphous matrix [52,57]. The non-collapsed glassy systems are more porous then rubbery systems and thus they allow easier diffusion of oxygen. Conversely, at T > Tg, collapse of dried materials occurs and it is associated with the disappearance of micropores and cavities, which limits oxygen access to oxygen-sensitive targets [41].

Overall, kinetic data on the degradation of oxygen-sensitive compounds such as β-carotene in dried matrices are not “generally valid”, but they are relative to the specific aw and Tg of the matrix. Moreover, when dried matrices are in contact with an environment with a given relative humidity, they come to the equilibrium aw through absorption or desorption of water [25]. This change in the aw and consequently in the Tg is likely to affect the rate of oxidative reactions. The description of the efficacy of β-carotene encapsulation technologies in dried system should therefore include the study of β-carotene stability over a wide aw and Tg range, in order to address the complexity of the relationships between moisture and stability. Hence, in the following paragraphs the kinetic data for β-carotene degradation are discussed with specific reference to the aw and Tg of the matrix. When these thermodynamic data were not available, the efficacy of the matrix system on β-carotene protection could not be fully demonstrated.

### 3.3. Rate of β-Carotene Degradation in Carrot Powder

The effect of aw on the stability of total carotenoids (α-carotene, β-carotene and lutein) in carrot powder was found to resemble the stability map observed for unsaturated lipids [58]. In fact, in dehydrated carrots, stored in the aw range 0.0–0.73, the rate of total carotenoid degradation as a function of aw showed a U-shaped curve and carotenoids were more stable in the aw range 0.32–0.57 [59]. Accordingly, in a following study the rate of β-carotene degradation in freeze-dried carrots was found to be at a minimum over the aw range 0.31–0.54, which was close to the GAB monolayer, with a half-life of 11–14 d at 40 °C. Below and above this aw range, β-carotene stability decreased markedly, with a half-life of 7, 8 and 3 d at aw 0.10, 0.22 and 0.75, respectively [60] (Table 1). Considering the activation energy values reported previously [21] (Table 2), the half-life was calculated at 25 °C using the Arrhenius equation and found to be low, i.e., 9, 12 and 16 d at aw 0.10, 0.22 and 0.31, respectively. The possibility to predict carotenoid stability in carrots based on Tg was investigated. A differential scanning calorimetry study of carrot powder revealed the existence of two Tg. The first Tg increases from −41.84 to 40.27 with decreasing aw from 0.75 to 0.11 and depends mainly on the presence of small molecules, such as sugars, organic acids and amino acids. The second Tg increases from 28.65 to 64.69 °C with decreasing aw from 0.75 to 0.11 and is associated to high molecular weight compounds, namely, starch, fibers and a small amount of proteins [61]. These data explain the low stability of carotenoids in carrot powder at aw 0.75 at 40 °C, because the difference between storage temperature and Tg is maximum. On the other hand, the low stability of carotenoids at aw 0.11 is not coherent with the high Tg associated to this aw value. Other factors are likely involved in poor stability, such as the presence of micropores in the glassy structure that favor the contact between air and solids, as observed in model glassy systems entrapping carotenoids [41].

### 3.4. Rate of Encapsulated β-Carotene Degradation in Dried Systems

A model polymeric matrix was designed to study the relationship between β-carotene stability, aw and Tg. In fact, β-carotene was suspended in aqueous solution of polyvinyl pyrrolidone (PVP-40) and gelatin (encapsulation efficiency was not provided), frozen in liquid nitrogen and then freeze-dried at −110 °C and 4 × 10^−4^ mbar. The powder obtained was equilibrated at different aw in the aw range 0.11–0.75 to investigate storage stability at 25 °C, but the microstructure of the powder and particle size were not specified [62]. The samples at aw 0.11–0.53 were in the glassy state, while those stored at aw 0.64 and 0.75 were in the supercooled state and showed structural collapse with a rather sticky or compacted macroscopic appearance. The degradation rate constants for β-carotene were maximum in the glassy state (below Tg), where β-carotene was effectively encapsulated, but the high porosity matrix allowed oxygen diffusion and then a fast degradation, with a half-life lower than 6 d at 25 °C. Oppositely, the lower degradation rate constants were observed at aw 0.64 and 0.75, with half-lives of about 66 and 99 d, respectively. However, under these conditions the matrix was collapsed; indeed, the matrix was fully plasticized and the structural collapse was supposed to cause the disappearance or the decrease of micropores, through which oxygen could enter or move in the matrix [62].

Other studies have investigated the development of molecular complexes to stabilize β-carotene. The inclusion agent cyclodextrins were proposed, since cyclodextrins are cyclic oligosaccharides with a hydrophobic cavity for holding hydrophobic guest molecules and a hydrophilic exterior for solubilization. However, theoretical investigations suggested that the incorporation of β-carotene in β-cyclodextrins is unstable [76]. Hence, in a following approach, two cross-linkers, epiclon and hexamethylene diisocyanate, were used to stabilize inclusion complex of β-cyclodextrins with β-carotene. Water suspensions of β-carotene and the derivatized cyclodextrins or not-derivatized cyclodextrin were agitated at high speed, followed by freeze drying at −86 °C and 4 × 10^−2^ bar [63]. Encapsulation efficiency was 44% in the absence of crosslinkers and 61% in the presence of crosslinkers. Porous nanosponges were obtained with average particle size of 654 nm for the epiclon crosslinker cyclodextrin complex and 590 nm for the hexamethylene diisocyanate crosslinkers cyclodextrin complex. On the other hand, the aw and Tg of the complexes were not reported. The stability of the inclusion complexes was evaluated at 25 °C and results showed that both crosslinked and not-crosslinked inclusion complexes provided similar protection to β-carotene, but the half-life was only approximately 5 d [63].

Amylose also forms single-helical inclusion complexes with various hydrophobic ligands. As a novel method to encapsulate and stabilize β-carotene, ternary complexes made of β-carotene, ascorbyl palmitate and amylose were developed, where ascorbyl palmitate acted as both as emulsifier and an antioxidant food additive. A suspension of water and dimethyl sulfoxide containing β-carotene, amylose and ascorbyl palmitate was stirred to form the inclusion complexes and then dried at room temperature. β-carotene showed relatively high stability upon storage at 25 °C, with a half-life of 26 d [64]. Hence, this approach allowed the better stability of ß-carotene with respect to the natural matrix carrot [60]. On the other hand, the encapsulation efficiency, the microstructure of the powder, aw and Tg of the complexes were not reported.

O/W emulsions are commonly used for lipophilic bioactive compounds encapsulation [77]. In one study, O/W emulsions of β-carotene in sunflower oil were dispersed in maltodextrin systems with dextrose equivalents (DE) 6, DE 11, and DE 25.5, frozen prior to freeze-drying at −80 °C and < 0.1 mbar for 48 h (encapsulation efficiency was not provided) [65]. The DE of maltodextrins is inversely related with their Tg and hence with the viscosity of the solution in which they are dissolved [78]. Therefore, maltodextrin DE 6, DE 11 and DE 25.5 are expected to display different behavior. The freeze-dried emulsions had particle size in the range 2–5 µm, with the highest value for the emulsion obtained with maltodextrins DE 25.5. The stability of the powders was investigated at aw 0.00, 0.33 and 0.75, at 24 °C. For all carriers, the highest stability was found at aw 0.75, i.e., above Tg, where transition of the systems to the rubbery state occurred and increased the size of oil droplets in the range 4–7 µm, while the highest value for the emulsion obtained with maltodextrins with DE 25.5 [65]. As a consequence, using maltodextrin DE 25.5 led to a half-life of 50, 69 and 99 d at aw of 0.00, 0.35 and 0.75, respectively. Interestingly, this system achieved was much higher β-carotene stability than that observed in dried carrots. The results also confirmed that structural collapse of the rubbery systems increased β-carotene stability. Moreover, the increased size of lipid particles as well as thicker encapsulant matrix membranes were found to enhance the stability of dispersed β-carotene in freeze-dried solids probably die to higher barrier properties toward oxygen diffusion [65].

Compared to the O/W emulsions obtained with maltodextrins DE 25.5, the use of Arabic gum or almond gum for O/W emulsification of β-carotene dissolved in sunflower oil followed by freeze-drying turned out to be less effective [66], although it was more effective than the natural matrix, i.e., carrot. The emulsifying properties of Arabic gum depend on 2% of protein contaminants among its main components that are polysaccharide [79]. Almond gum also contains 2.45% of protein, which contributes to its emulsifying properties [80]. Almond gum exhibited emulsifying properties comparable to Arabic gum, but it displayed slower dynamics of adsorption and reorganization at the oil/water interface [81]. In these systems, O/W emulsions of β-carotene were frozen at −30 °C and freeze-dried at −120 °C and 0.3 × 10^−4^ mbar for 48 h, with secondary drying at 25 °C [66]. The encapsulation efficiency ranged between 93.43 and 91.82% for almond gum and Arabic gum, respectively. The average diameter of the powders was 3.7 and 5.1 µm for almond gum and Arabic gum, respectively. The retention of β-carotene encapsulated with almond gum or Arabic gum was investigated during storage at 25 °C and the resulting half-life for β-carotene was 28 and 40 d at aw 0.1 for Arabic gum and almond gum, respectively. Increasing aw to 0.45 resulted in decreased β-carotene half-life in both systems, but increased β-carotene half-life was observed at aw 0.8 when the systems were collapsed.

The use of Arabic gum or almond gum for O/W encapsulation of β-carotene dissolved in sunflower oil, previously obtained by freeze-drying was also applied by spray-drying (diameter nozzle 0.5 mm, pressure of compressed air 2 bar, inlet temperatures were maintained at 180 °C, feed rate was 20 mL/min) [67]. In this system, encapsulation efficiency of 66.4 and 69.8% were observed for almond gum and Arabic gum, respectively, which are lower than those observed upon freeze drying [66,67]. The average diameter of the powders was 2.1 and 3.2 µm for almond gum and Arabic gum, respectively, which are lower than those obtained by freeze-drying. When Arabic gum was applied, during storage at aw 0.1 at 25 °C, the half-life of β-carotene was 72 d; then it decreased to 23 d when aw increased from 0.10 to 0.45. Hence, higher stability was observed for the spray-dried system compared to the freeze-dried system [66,67]. As expected, at aw 0.80 β-carotene stability increased due to the occurrence of glass transition, causing structural collapse and a decrease in the micropores of the matrix. When almond gum was used in the place of Arabic gum, lower stability was found and no difference was observed between the spray-dried and freeze-dried system [66,67].

The development of W_1_/O/W_2_ emulsions was also proposed for encapsulation of lipophilic compounds [82]. However, for β-carotene W_1_/O/W_2_ emulsions did not lead to great improvement of stability. In fact, β-carotene was dissolved into a commercial blend of sunflower canola–cartamum oils to build the lipid phase, which was emulsified with a solution of gellan gum added with both hydrophilic emulsifiers (esters of monoglycerides and diglycerides of diacetyl tartaric acid) and hydrophobic emulsifiers (esters of polyglycerol polyricinoleate fatty acids). Then, the primary emulsion was re-emulsified in the aqueous solutions of the biopolymers Arabic gum and maltodextrin DE 10 and then spray-dried (20 mL/min, 2.8 bar air pressure, inlet temperature of 170 °C) [68]. The ratios among various emulsion components were optimized to achieve a microencapsulation efficiency of 87.5%. The particle size was 34 µm. The emulsions were equilibrated at aw levels in the range 0.022–0.845, in order to investigate storage stability at 35 °C. β-carotene degradation kinetics was practically the same in the aw range 0.022 to 0.515, where it displayed a half-life of 21 d. However, the degradation rate surged to a maximum at aw of 0.628, which could be due to pro-oxidant effects caused by (a) reduced viscosity promoting mobility, (b) dissolution of precipitated catalysts, and/or (c) swelling of the solid matrices exposing new catalytic surfaces. The glass transition phenomenon was not investigated but stability was correlated to structural modifications. Indeed, in the aw range 0.742 to 0.821, β-carotene degradation rate fell to a minimum constant value, probably due to the formation of a new gel-like compact structure in which β-carotene was reincorporated in the biopolymer matrix [68].

One strategy to increase β-carotene stability is to strengthen the interface of the emulsion by applying multilayers of polymers. Moreover, using high concentrations of hydrophilic solids in the emulsion to be dried usually leads to a high bulk density and, hence, a low amount of occluded air in the powder [69,70]. A comparison was made between single-layer and layer-by-layer (LBL) emulsions designed to encapsulate β-carotene and lutein dissolved in sunflower oil. Single-layer emulsions were obtained using sunflower oil as the oil phase, whey protein isolate as emulsifiers and trehalose as wall material, while LBL emulsions were obtained by adding Arabic gum as the second layer [69]. In fact, whey proteins at a low pH can be covered by negatively charged polyelectrolytes, including Arabic gum, to give improved resistance of emulsions against freeze–thaw cycles, lipid oxidation and high salt concentrations. Trehalose was used as a hydrophilic component that can form a continuous solid phase of the dried formulation, though, on the other hand, the bulk density was not measured. The single layer and LBL emulsions were frozen at −80 °C, freeze-dried at pressure < 0.1 mbar for at least 72 h, but the encapsulation efficiency was not reported. β-carotene degradation was found to follow two-step first-order kinetics of degradation; the first step was faster and was attributed to the degradation of β-carotene dissolved in the surface oil, while the second step was attributed to the degradation of encapsulated oil. At 37 °C, the system was not collapsed. Interestingly, the half-life for β-carotene in both the LBL system and the single-layer system at aw 0.33 and 37 °C were approximately 30 and 49 d, respectively, which are relatively high and could be attributed to the protecting effect of trehalose [69]. The same freeze-dried β-carotene encapsulation systems were also studied at aw 0.14 and compared to similar systems obtained by spray-drying (inlet temperature 185 °C). For spray-drying, however, maltodextrin DE 23–27 was also included as wall material. Interestingly, the freeze-dried sample showed a relatively high stability at aw 0.14, as already observed for aw of 0.33 [70], with a half-life higher than 78 d at 35 °C. The spray-dried samples were even more stable than the freeze-dried samples, with half-lives higher than 99 d. This effect was attributed to less air occluded in the spray-dried emulsions due to the presence of trehalose [70].

Another approach to improve the barrier that protects β-carotene is to use combination of polysaccharides and proteins. A relevant increase in β-carotene stability in the glassy state was achieved when β-carotene and α-carotene were emulsified in O/W palm oil emulsion containing tocopherols and tocotrienols, maltodextrin DE 10, sodium caseinate and soy lecithin and then encapsulated by either solution-enhanced dispersion by supercritical carbon dioxide (SEDS) (125 bar, 50 °C, CO_2_ feed 150 L/h) or spray-drying (feed rate 15 mL/min; inlet temperature 165 °C) [71]. In fact, casein arrangement of hydrophobic and hydrophilic amino acid residues makes it an ideal emulsifier [83]. SEDS-produced powder had an aw of 0.47 and spray-dried powder had an aw of 0.40 (at 26.6 °C). Encapsulation efficiency was 92 and 79.3%, respectively. The average diameter was 5.8 and 16.6 for SEDS-obtained powder and spray-dried powder, respectively. Both the SEDS and spray-drying techniques increased the storage stability of carotenoids with respect to the free oil. Examination of the inner structure of microcapsules produced by both SEDS and spray-drying methods provided some insight into the property differences: microcapsules produced with the SEDS had many fine pores that contained oil or CO_2_ gas, whereas those produced with the spray-drying method had heavy walls and large voids that contained oil and air [71]. The degradation rate for β-carotene was much lower than those found in previous studies cited above, with half-lives of 149 and 199 d for the spray-drying and SEDS techniques, respectively. This effect may be due to the combined presence of polysaccharides and protein in the wall material, that could provide a better protection then the separated polymers. Indeed, in a previous study on a system made with whey protein and pectin it was demonstrated that during heat drying, Maillard reaction occurs, leading to increased stability of the encapsulated β-carotene [71]. Moreover, the protection of β-carotene by tocopherols and tocotrienols cannot be ruled out.

Other combinations of polysaccharides with protein were investigated. Pullulan was used in combination with whey protein isolate to encapsulate β-carotene dissolved in medium-chain triacylglycerol oil by a pre-emulsification step followed by electrospraying (feed flow rate: 0.01 mL/min; voltage: 18 kV, temperature: 20 °C) [72]. In fact, in a previous study, pullulan alone displayed good film-forming properties but low emulsifying properties, resulting in low encapsulation efficiency [84]. Hence, whey proteins were used to improve the emulsifying properties [72]. The encapsulation efficiency was only 53%. The average particle size of the powder was 0.54 µm. β-carotene degradation was modelled with Avrami’s equation and was shown to follow diffusion limited model for aw 0.2 and first-order kinetic model for aw 0.4 and 0.6. From the equations provided, it could be calculated that the half-life of β-carotene was 46, 81 and 86 d at room temperature and aw of 0.6, 0.4 and 0.2, respectively. The lower stability of the nanocapsules at higher aw was attributed to pullulan dissolution [72].

In another approach, the emulsifier was functionalized to advance its performance. N-octenyl succinate anhydride (OSA)-modified starches, such as the commercial HI-CAP, CAPSUL and CAPSUL TA, have been developed to improve emulsifying and film-forming properties, low viscosities, high oil-loading capacities and oxygen barrier properties [85]. Hence, these compounds were applied with medium-chain triacylglycerol oil to form β-carotene O/W nanoemulsions, with average particle size in the range 0.10–0.15 µm depending on the modified starch used. These latter O/W nanoemulsions were spray-dried (air inlet temperature of 190 °C, feed rate of 20 mL/min) but the encapsulation efficiency was not reported. β-carotene loss was studied during storage in the aw range 0.11–0.97 at 25 °C for 30 d. Differently from most of the other studies considered so far, which applied a first-order kinetics, β-carotene degradation was studied with the Weibull model. For the samples at aw ≤ 0.75, the shape values were greater than 1 which indicates that the general shape of the degradation curve is convex with a fast degradation. When aw was 0.97, the shape value was less than 1 or close to 1, which indicated that the degradation curve is concave with the highest loss of β-carotene during the initial stage of storage [73]. Among the different modified starches, β-carotene degradation was minimum in HI-CAP and maximum in CAPSUL: this trend was associated to the oxygen permeability of the modified starches, which was lower for HI-CAP than for CAPSUL and CAPSUL TA [73]. In particular, β-carotene encapsulated in HI-CAP showed high stability in the glassy state, with a half-life of 53 d at aw 0.11 and 25 °C, which could be due to both the oxygen barrier properties of HI-CAP and to the smooth surface of the powder that was found to be more compact than the powders obtained with the other modified starches. In the powders obtained with CAPSUL and CAPSUL TA, the stability was very low, below an aw of 0.75, but the degradation rate sharply decreased at aw 0.97, where the material was collapsed [73], thus confirming previous findings [62].

Another strategy to increase β-carotene stability is to co-encapsulate an antioxidant compound. To this aim, β-carotene was dissolved in flaxseed oil with eugenol and mixed with two types of OSA starches differing for the molecular weight (MW 9.4 × 10^5^ or MW 5.3 × 10^6^), passed through a microfluidizer and the spray-dried (feed rate: 10 mL/min, inlet temperature: 170 °C) [74]. The encapsulation efficiency was approximately 90% in both systems. The aw was below 0.26 and Tg was not reported, but the systems likely were in the glassy state. The particle size was found to be 0.126 and 0.241, with the highest value found for the system with the highest MW OSA starch. Upon storage at 40 °C, it was observed that eugenol was effective in protecting β-carotene from degradation, with the half-life increasing from 21 d in absence of eugenol to more than 30 d in presence of eugenol. The molecular weight of OSA starch was also important; in fact, lower MW starch provided better protection. This effect was attributed to tight adsorption of emulsifier at the interface, lower viscosity, reduced entrapment of air in emulsion feed for spray drying and finally formation of a stable film with high viscoelasticity that prevented the oxidation of core material [74].

In a following approach, multiple strategies were combined together to enhance protection of β-carotene. In fact, a high concentration of a hydrophilic solid compound, i.e., sucrose, was dissolved in the emulsion to be dried as proposed previously [69]. Moreover, antioxidants were added in both the hydrophilic phase and the lipid phase. To this aim, β-carotene and α-tocopherol were dissolved in corn oil and then homogenized at high pressure with a solution containing OSA starch, ascorbic acid and various amounts of sucrose before spray-drying (inlet temperature at 170 °C; air pressure of the atomizer 0.4 MPa, feed rate 500–1500 mL/h, inlet hot air rate 50–300 m^3^/h). [75]. The surface oil was less than 5% and slightly decreased with increasing sucrose content. With an increasing ratio of sucrose, the average size of the emulsion droplets decreased. For instance, when the OSA: sucrose ratio was 1:1, the average size was 0.25 µm. The aw was 0.26 whatever the sucrose content, while the Tg of the wall material decreased with increasing sucrose content, even if in any case it was well above room temperature, indicating that the dried O/W emulsions were in the glassy state. The dried emulsions were stored for 30 d at 60 °C and found to be very stable. For instance, when OSA: sucrose ratio was 1:1, the half-life for β-carotene degradation was higher than 30 d at 60 °C, suggesting that the dried emulsions would be very stable at room temperature [75].

### 3.5. Activation Energy for β-Carotene Degradation

Few studies have investigated the activation energy for β-carotene degradation (Table 2). In carrot, the activation energy for β-carotene degradation was found to depend slightly on the aw level, with values ranging from about 16 to about 18 kJ/mol at aw of 0.10 and 0.30, respectively [21]. From the kinetic data describing β-carotene degradation in room-temperature-dried molecular complexes made with amylose and ascorbyl palmitate, an activation energy of about 23 kJ/mol was calculated using the Arrhenius equation [64]. In spray-dried emulsions or emulsions dried by SC-CO_2_, made with palm oil-containing tocopherols and tocotrienols, maltodextrins DE 10, sodium caseinate and soy lecithin activation energy was found to be 25 and 29 kJ/mol, respectively [71]. Since these latter systems only differed for the microstructure, this result seems to indicate that the microstructure may slightly affect activation energy.

Spray-died systems made with sunflower oil, trehalose, whey protein and maltodextrins MD 23–27, with or without a second layer of Arabic gum, showed that the activation energy for β-carotene degradation was different in the temperature range below Tg and above Tg [70], suggesting that the viscosity of the matrix and its porosity also affects the activation energy, probably affecting heat transfer. Moreover, the freeze-dried systems obtained with the same ingredients (without maltodextrins) had higher activation energies that those of the spray-dried systems, which differed in the temperature range below Tg and above Tg, confirming that the microstructure of the matrix affects heat transfer kinetics [69]. As a general conclusion, beside the role of the ingredients of the formulations, the impact of the microstructure on activation energy seem to be relevant and needs further investigation.

## 4. Conclusions and Future Perspectives

Considering a dried natural matrix such as carrot powder, little protection of β-carotene occurs during storage, with a half-life below 16 d at 25 °C and minimal stability at low aw, where the system is in the glassy state. Despite the low molecular mobility of the glassy state, small molecules such as oxygen can diffuse in the matrix, promoting oxidation, probably due to the porous structure of the glassy system. The development of micro- and nano-structures for β-carotene stabilization is challenging. One strategy to increase the stability of β-carotene in the glassy state was to apply high concentrations of hydrophilic solids (trehalose or sucrose) in the mixture to be dried, which led to a low amount of occluded air in the powder. The use of antioxidant compounds having various polarities, such as ascorbic acid, eugenol and tocopherol, co-encapsulated in the micro- and nano-structures, was also successful to decrease the degradation rate of β-carotene. Moreover, a promising approach was to increase the performance of the interface between the air and oil phase in which β-carotene was dissolved, by using multilayer biopolymers, proteins linked to polysaccharides via the Maillard reaction or functionalized biopolymers such as OSA modified starches. By these approaches, the half-life of β-carotene could increase up to about 200 d at 25 °C.

Future research needs to extend data on the activation energy and the effect of aw on β-carotene degradation, in order to accurately predict the stability of the designed structures upon environmental temperature and humidity fluctuations. Moreover, advance knowledge on oxygen permeability across various interfaces, as affected by temperature, aw and Tg of the system, could lead to the selection of the most appropriate biopolymers for β-carotene protection and to better explore the potential of the wide array of natural biopolymers as barrier materials for β-carotene encapsulation.

## Figures and Tables

**Table 1:** Dried systems for the delivery of β-carotene: matrix ingredient, structure, average particle size (PS, µm), water activity (aw), glass transition temperature (Tg, °C), degradation rate constants (k × 10^3^, d^−1^) and half-life (t_1/2_, d) for β-carotene during storage in the dark at a given temperature (°C).

Matrix Ingredients	Matrix Structure	PS	T	aw	Tg	k × 10^3^	t_1/2_	Ref.
carrot	FD powder	n.d. n.d. n.d. n.d. n.d	40	0.10 0.22 0.31 0.53 0.75	40.64 13.45 −11.44 −20.38 −41.29	104 ^a^ 82 ^a^ 62 ^a^ 48 ^a^ 271 ^a^	7 8 11 14 3	k [60] Tg [61]
β-carotene, polyvinyl pyrrolidone −40 and gelatin	FD molecular complexes	n.d. n.d n.d.	25	0.11 0.44 0.53	86 50 25	270 ^b^ 220 ^b^ 110 ^b^	2 3 6	[62]
	collapsed molecular complexes	n.d. n.d		0.64 0.75	7 −9	10 ^b^ 7 ^b^	69 99	
β-carotene, β-cyclodextrins/epiclon	FD molecular complexes	0.65	25	n.d.	n.d.	n.d.	5	[63]
β-carotene, β-cyclodextrins/hexamethylene diisocyanate	FD molecular complexes	0.59		n.d.	n.d.	n.d.	5	
β-carotene, amylose, ascorbyl palmitate	RT molecular complexes	n.d.	20	n.d.	n.d.	32.8 ^a^	26	[64]
β-carotene, sunflower oil, maltodextrins DE 4	FD O/W emulsion	~2 ~2	24	0.00 0.35	n.d. n.d.	~16 ^a^ ~14 ^a^	~43 ~50	[65]
	collapsed O/W emulsion	~2.5		0.75	n.d.	~12 ^a^	~58	
β-carotene, sunflower oil, maltodextrins DE 10	FD O/W emulsion	~3 ~3		0.00 0.35	n.d. n.d.	~18 ^a^ ~18 ^a^	~39 ~39	
	collapsed O/W emulsion	~4		0.75	n.d.	~10 ^a^	~69	
β-carotene, sunflower oil, maltodextrins DE 25.5	FD O/W emulsion	~4 ~4		0.00 0.35	n.d. n.d.	~14 ^a^ ~10 ^a^	~50 ~69	
	collapsed O/W emulsion	~7		0.75	−28	~7 ^a^	~99	
β-carotene, sunflower oil, Arabic gum	FD O/W emulsion	5.1 n.d.	25	0.10 0.45	n.d. n.d.	24 ^a^ 39.7 ^a^	28 17	[66]
	collapsed O/W emulsion	n.d.		0.80	n.d.	n.d.	>70	
β-carotene, sunflower oil, Almond gum	FD O/W emulsion	3.7 n.d.		0.10 0.45	n.d. n.d.	17.8 ^a^ 25 ^a^	40 28	
	collapsed O/W emulsion	n.d.		0.80	n.d.	n.d.	>70	
β-carotene, sunflower oil, Arabic gum	SD O/W emulsion	3.2	25	0.10 0.45	n.d. n.d.	9.6 ^a^ 30 ^a^	72 23	[67]
	collapsed O/W emulsion	n.d.		0.80	n.d.	n.d.	n.d.	
β-carotene, sunflower oil, Almond gum	SD O/W emulsion	2.1		0.10 0.45	n.d. n.d.	25 ^a^ 45 ^a^	28 15	
	collapsed O/W emulsion	n.d.		0.80	n.d.	n.d.	n.d.	
β-carotene, sunflower canola-cartamum oils, gellan gum, Arabic gum, maltodextrins DE 10, mono- and di-glycerides, diacetyl tartaric acid and polyglycerol polyricinoleate	SD W_1_/O/W_2_ emulsion	34	35	0.02–0.51	n.d.	23.5 ^a^	21	[68]
	dissolved emulsion	n.d.		0.63	n.d.	160 ^a^	4	
	gel-like structure	n.d.		0.74–0.82	n.d.	20 ^a^	35	
β-carotene, sunflower oil, trehalose, whey protein	FD O/W emulsion	n.d.	37	0.33	n.d.	~23 ^a^	~30	[69]
β-carotene, sunflower oil, trehalose, whey protein, Arabic gum	FD LBL O/W emulsion	n.d.		0.33	n.d.	~14 ^a^	~49	
β-carotene, sunflower oil, trehalose, whey protein	FD O/W emulsion	n.d.	35	0.14	50	n.d.	>78	[70]
β-carotene, sunflower oil, trehalose, whey protein, Arabic gum	FD LBL O/W emulsion	n.d.		0.14	50	n.d.	>78	
β-carotene, sunflower oil, trehalose, maltodextrin DE 23–27, whey protein	SD O/W emulsion	n.d.		0.16	64	n.d.	>99	
β-carotene, sunflower oil, trehalose, maltodextrin DE 23–27, whey protein, Arabic gum	SD LBL O/W emulsion	n.d.		0.17	64	n.d.	>99	
β-carotene, palm oil, tocopherols and tocotrienols, maltodextrins DE 10, sodium caseinate and soy lecithin	SEDS O/W emulsion	5.8	25	0.47	n.d.	4.65 ^a^	149	[71]
	SD O/W emulsion	16.6		0.40	n.d.	3.48 ^a^	199	
β-carotene, medium chain triglycerides, pullulan, whey protein	ED O/W emulsion	0.54 n.d. n.d.	25	0.2 0.4 0.6	n.d n.d. n.d.	n.d. n.d. n.d.	86 81 46	[72]
β-carotene, medium chain triglycerides, OSA modified starch HI-CAP	SD O/W emulsion	0.15 n.d. n.d. n.d.		0.11 0.33 0.52 0.75	n.d. 31 9 −26	13(1.2) ^c^ 14(1.2) ^c^ 34(1.5) ^c^ 25(1.4) ^c^	50 46 17 24	[73]
	collapsed emulsion	n.d.		0.97	n.d.	18(1.0) ^c^	39	
β-carotene, medium chain triglycerides, OSA modified starch CAPSUL	SD O/W emulsion	0.10 n.d. n.d. n.d.		0.11 0.33 0.52 0.75	n.d. 80 73 55	34(1.2) ^c^ 39(1.2) ^c^ 41(1.2) ^c^ 44(1.2) ^c^	19 17 16 15	
	collapsed O/W emulsion	n.d.		0.97	n.d.	21(0.8) ^c^	36	
β-carotene, medium chain triglycerides, OSA modified starch CAPSUL TA	SD O/W emulsion	0.11 n.d. n.d. n.d.		0.11 0.33 0.52 0.75	n.d. 83 75 56	33(1.2) ^c^ 35(1.3) ^c^ 38(1.3) ^c^ 41(1.3) ^c^	20 18 16 15	
	collapsed O/W emulsion	n.d.		0.97	n.d.	19(1.0) ^c^	36	
β-carotene, flaxseed oil, eugenol, OSA starch (MW 9.4 × 10^5^)	SD O/W emulsion	0.13	40	0.28	n.d.	n.d.	>30	[74]
β-carotene, flaxseed oil, eugenol, OSA starch (MW 5.3 × 10^6^)	SD O/W emulsion	0.24		0.19	n.d.	n.d.	21	
β-carotene, α-tocopherol, corn oil, OSA starch, ascorbic acid, sucrose	SD O/W emulsion	0.25	60	0.26	83	n.d.	>30	[75]

Rate constant and half-life refer all-*trans*-β-carotene degradation. ^a^ First-order kinetic model. ^b^ Fractional conversion model. ^c^ Weibull model, with the shape value n indicated in brackets. DE, dextrose equivalents; OSA, N-octenyl succinate anhydride; O, oil; W, water; LBL, layer-by-layer; FD, freeze-dried; RT, room-temperature dried; SD, spray-dried; SEDS, supercritical CO_2_. enhanced dispersion dried; ED, electro-sprayed; n.d., not determined.

**Table 2:** Activation energy (Ea, kJ/mol) in the given temperature range (T range, °C) and at a specific aw for the degradation of β-carotene encapsulated in dried micro- and nano-structures.

Matrix Ingredients	Matrix Structure	T Range	aw	Ea	Ref.
carrot	AD powder	60–80	0.10 0.20 0.30	15.69 16.27 17.61	[21]
β-carotene, amylose, ascorbyl palmitate	RT molecular complexes	20–30	n.d.	23 (calculated)	[64]
palm oil containing tocopherols and tocotrienols, maltodextrin DE 10, sodium caseinate and soy lecithin	SD O/W emulsion	25–85	0.40	29	[71]
palm oil containing tocopherols and tocotrienols, maltodexrtin DE 10, sodium caseinate and soy lecithin	SEDS O/W emulsion	25–85	0.47	25	
β-carotene, sunflower oil, trehalose, whey protein	FD O/W emulsion	25–45 45–65	0.14 0.14	58.29 (below Tg) 16.81 (above Tg)	[70]
β-carotene, sunflower oil, trehalose, whey protein, Arabic gum	FD LBL O/W emulsion	25–45 45–65	0.14 0.14	29.72 (below Tg) 69.83 (above Tg)	
β-carotene, sunflower oil, trehalose, maltodextrin DE 23–27, whey protein	SD O/W emulsion	25–45 45–65	0.16 0.16	23.59 (below Tg) 16.88 (above Tg)	
β-carotene, sunflower oil, trehalose, maltodextrin DE 23–27, whey protein, Arabic gum	SD LBL O/W emulsion	25–45 45–65	0.17 0.17	12.71 (above Tg) 28.34 (below Tg)	

Activation energy refers to refer all-*trans*-β-carotene degradation: DE, dextrose equivalents; O, oil; W, water; LBL, layer-by-layer; FD, freeze-dried; RT, room temperature dried; AD, air-dried; SD, spray-dried; SEDS, supercritical CO_2_ enhanced dispersion dried; n.d., not determined.
